# OmpR, a response regulator of the two-component signal transduction pathway, influences *inv* gene expression in *Yersinia enterocolitica* O9

**DOI:** 10.3389/fcimb.2012.00153

**Published:** 2012-12-18

**Authors:** Marta Brzóstkowska, Adrianna Raczkowska, Katarzyna Brzostek

**Affiliations:** Department of Applied Microbiology, Institute of Microbiology, Faculty of Biology, University of WarsawWarsaw, Poland

**Keywords:** invasin, OmpR regulator, RovA regulator, signal transduction pathway, *Yersinia enterocolitica*

## Abstract

The environmental control of invasin (*inv*) expression in *Yersinia enterocolitica* is mediated by a regulatory network composed of negative and positive regulators of *inv* gene transcription. Previously, we demonstrated that OmpR, a response regulator of the two-component signal transduction pathway EnvZ/OmpR, negatively regulates *inv* gene expression in *Y. enterocolitica* O9 by direct interaction with the *inv* promoter region. This study was undertaken to clarify the role of OmpR in the *inv* regulatory circuit in which RovA protein has been shown to positively regulate *inv* transcription. Using *ompR*, *rovA*, and *ompR rovA Y. enterocolitica* mutant backgrounds we showed that the inhibitory effect of OmpR on *inv* transcription may be observed only when RovA is present/active in *Y. enterocolitica* cells. To extend our research on *inv* regulation we examined the effect of OmpR on *rovA* gene expression. Analysis of *rovA-lacZ* transcriptional fusion in *Y. enterocolitica* wild-type and *ompR* background indicated that OmpR does not influence *rovA* expression. Thus, our results indicate that OmpR influences *inv* expression directly via binding to the *inv* promoter, but not through modulation of *rovA* expression.

## Introduction

*Yersinia enterocolitica* is a human gastrointestinal pathogen that is able to exist free-living in the environment. Survival in the different ecological niches requires adaptation of pathogen to the changing physico-chemical conditions, such as temperature, pH, osmolarity, accessibility of nutrients, viscosity of the medium, etc. (Straley and Perry, 1995; Bottone, 1997).

The molecular mechanisms enabling bacterial response to signals coming from the external environment are very complex and involve two-component transduction systems (TCSs) (Stock et al., 1989; Hoch and Silhavy, 1995). TCSs play a significant regulatory role in the transduction of environmental signals in various species of bacteria, including the genus Yersinia. Based on the entire genome sequence of *Y. enterocolitica* and theoretical relationships, a number of putative TCSs of *Y. enterocolitica* have been identified, however, the function of most of them still requires experimental verification (Marceau, 2005). The *in silico* analysis of the genome of *Yersinia pestis* (strain CO92) has revealed 29 putative TCSs (including 4 pseudogenes), whereas the genome of the enteropathogenic *Yersinia pseudotuberculosis* appears to encode 24 complete TCSs (Flamez et al., 2008; O'Loughlin et al., 2010).

One of the most extensively studied TCSs is the EnvZ/OmpR regulatory system of non-pathogenic *Escherichia coli* (*E. coli*) K-12 that controls the expression levels of outer membrane porin proteins OmpF and OmpC in response to changes in the osmolarity of the environment (Russo and Silhavy, 1991; Cai and Inouye, 2002). The basic components of EnvZ/OmpR transduction pathway are a dimeric histidine kinase EnvZ, serving as a signal sensor, and its cognate response regulator OmpR, a cytoplasmic winged helix transcription factor (Forst and Roberts, 1994; Kenney, 2002). Upon sensing a signal, the EnvZ autophosphorylates and then the phosphate group of EnvZ-P is transferred to OmpR to form phosphorylated OmpR (OmpR-P). Since EnvZ can act also as a phosphatase, it is able to remove the phosphoryl group from the phosphorylated/activated form of OmpR. In response to environmental changes, the ratio of the kinase to the phosphatase activity of EnvZ modulates the cellular level of OmpR-P (Yoshida et al., 2002; Qin et al., 2003). OmpR-P serves as a transcription factor which, by binding to promoter regions of target genes regulates their expression. OmpR protein has been revealed as a global transcriptional regulator implicated in the control of various cellular processes and functions in many Gram-negative bacteria (Gibson et al., 1987; Higashitani et al., 1993; Shin and Park, 1995; Jubelin et al., 2005).

It has also been shown that, OmpR plays a significant role in controlling the expression of virulence factors of bacterial pathogens (Dorman et al., 1989; Bernardini et al., 1990; Bang et al., 2000; Lee et al., 2000; Brzostek et al., 2007). In yersiniae, the mechanism of TCS action revealed in biochemical and genetic analyses seems to follow the same pattern as observed for EnvZ/OmpR in *E. coli*. Moreover, a recent comparative transcriptome analysis of *Y. pestis* identified a set of 224 genes affected by the *ompR* mutation leading to detailed studies of the OmpR-dependent expression of *ompC, F, X*, and *ompR* (Gao et al., 2011). It also appears that OmpR could operate as a global regulatory protein in *Y. enterocolitica* cells. The involvement of EnvZ/OmpR system of *Y. enterocolitica* in the regulation of porin synthesis was described (Brzostek et al., 1989; Brzostek and Raczkowska, 2007). The discovery that the *ompR* mutant of *Y. enterocolitica* serotype O8 is attenuated in the murine yersiniosis model (Dorrell et al., 1998) was an incentive to investigate the putative correlation between the EnvZ/OmpR functions and the expression of virulence genes in pathogenic *Y. enterocolitica* serotype O9 (Brzostek and Raczkowska, 2003). The *in vitro* analysis of the growth and survival of *Y. enterocolitica* O9 cells lacking the OmpR protein, subjected to various environmental stresses, revealed that OmpR is involved in the adaptation of *Y. enterocolitica* to high osmolarity, oxidative stress and low pH. Recent studies provided evidence that, OmpR is involved in the control of motility by positive regulation of flagellar master operon *flhDC* in both *Y. enterocolitica* O9 and *Y. pseudotuberculosis* (Hu et al., 2009b; Raczkowska et al., 2011b). This finding is in contrast to the negative role the regulator was shown to play in *E. coli* (Shin and Park, 1995). Lastly, it has been shown that OmpR-dependent regulation of biofilm is an additional aspect of OmpR regulatory function in *Y. enterocolitica* O9 (Raczkowska et al., 2011c).

In addition, we demonstrated previously that OmpR negatively regulates invasin (*inv*) gene expression in *Y. enterocolitica* O9 by direct interaction with the *inv* promoter region (Brzostek et al., 2007). *inv* is an important adhesion/invasion factor localized in the outer membrane of *Y. enterocolitica* and *Y. pseudotuberculosis*, which is responsible for the ability of enteropathogenic *Yersinia* to penetrate the epithelium of the host intestines (Grutzkau et al., 1990; Pepe and Miller, 1993; Isberg and Van Nhieu, 1995; Dersch and Isberg, 2000; Nagel et al., 2001). Various environmental factors such as temperature, growth phase, nutrients, pH, and osmolarity influence the *inv* gene transcription levels, thus modulating the expression of *inv*. The highest levels of *inv* expression in *Y. enterocolitica* O8 and *Y. pseudotuberculosis* were observed in the stationary phase cultures incubated at moderate temperature (23–26°C) (Pepe et al., 1994; Nagel et al., 2001). However, recent studies of the effect of temperature and growth phase on the *inv* gene expression using the *luxCDABE* reporter system showed differences in the *inv* promoter activity between strains of *Y. enterocolitica* serotype O8 and O9 (Trček et al., 2010).

Genetic and physiological studies have revealed that numerous regulatory proteins are involved in the process of modulation of *inv* expression in response to environmental cues. The thermoregulation of *inv* gene expression in *Y. enterocolitica* O8 and *Y. pseudotuberculosis* seems to involve at least three regulatory proteins, namely RovA, H-NS, and YmoA (Ellison et al., 2003; Heroven et al., 2004; Tran et al., 2005; Ellison and Miller, 2006b). RovA, a member of the large MarR/SlyA family of transcriptional regulators identified in *Enterobacteriaceae*, acts as a positive regulator of *inv* expression at low temperature (23–26°C) in both enteropathogenic yersiniae (Revell and Miller, 2000; Nagel et al., 2001; Ellison and Miller, 2006a). Moreover, RovA protein has been indicated as an important factor involved directly or indirectly in the transcriptional regulation of many other *Yersinia* genes, some of which have been linked to virulence (Ellison et al., 2004; Cathelyn et al., 2006, 2007). H-NS is a small nucleoid-associated protein identified as a repressor of *inv* expression (Atlung and Ingmer, 1997; Heroven et al., 2004; Ellison and Miller, 2006b). In *Y. enterocolitica*, repression of *inv* seems to be mediated not solely by H-NS. YmoA, a histone-like protein acts together with H-NS inhibiting the expression of *inv* gene (Ellison et al., 2003). Following the up-shift of temperature to 37°C, H-NS and YmoA are believed to form a transcriptional repression complex on the *inv* promoter, which becomes very effective in competing with the RovA protein for binding to the *inv* promoter region. At 26°C, higher levels of cellular RovA block the *inv* promoter binding sites, which antagonizes H-NS/YmoA-mediated repression leading to elevated *inv* expression (Ellison et al., 2003; Ellison and Miller, 2006b). Thermoregulation of RovA expression has been found to be a very complex process involving transcriptional and post-transcriptional mechanisms. It has been recently shown that the temperature shift from 25°C to 37°C not only affects *rovA* expression, but also RovA DNA-binding activity and renders the RovA protein more susceptible to degradation by the Lon and ClpP proteases (Herbst et al., 2009; Uliczka et al., 2011). Apart from the well-studied RovA and H-NS/YmoA interactions with *inv* promoter region in both enteropathogenic yersiniae, it has been also reported that the transcriptional regulator CpxR of the Cpx extracytoplasmic-stress-responsive TCS of *Y. pseudotuberculosis* modulates *inv* expression by direct negative effect on both *inv* and *rovA* transcription (Carlsson et al., 2007; Liu et al., 2011).

The results of our previous study revealed the involvement of the transcriptional regulator OmpR in the negative regulation of the *inv* gene in *Y. enterocolitica* O9 (Brzostek et al., 2007). Recently, the osmoregulation of *inv* expression was found to be a multifaceted process involving both OmpR-dependent and -independent mechanisms (Raczkowska et al., 2011a).

The aim of the present study was to more precisely define the function of OmpR in the transcriptional response of the *inv* gene. In light of the evidence for the participation of RovA in positive regulation of *inv* expression, we focused on the role of OmpR in the regulatory OmpR/RovA interplay.

## Materials and methods

### Bacterial strains, plasmids, and growth conditions

The bacterial strains and plasmids used in this study are listed in Table 1. *Y. enterocolitica* strains were cultivated in Luria-Bertani (LB) medium at 25°C or 37°C with aeration to mid-log-phase or to early stationary growth phase.

**Table 1:** **Strains and plasmids used**.

**Strain or plasmid**	**Description**	**Reference or source**
***Y. enterocolitica* O9**		
Ye9	pYV^+^, wild type	Laboratory collection
Ye9N	pYV^+^, Nal^R^	Brzostek et al., 2007
AR4	pYV^+^, Nal^R^, Δ*ompR*::*kan*	Brzostek and Raczkowska, 2003
AS3	pYV^+^, Nal^R^, *rovA*::pEP185.2, Cm^R^	This work
AC1	pYV^+^, Nal^R^, Δ*ompR*::*kan*, *rovA*::pEP185.2, Cm^R^	This work
YeR2	pYV^+^, Nal^R^, *rovA::lacZYA*, Cm^R^	This work
ARR8	pYV^+^, Nal^R^, Δ *ompR*::*kan*, *rovA::lacZYA*, Cm^R^	This work
***E. coli***		
S17-1 λ*pir*	*pro thi recA hsdR514* (R^+^M^−^) *λpir* RP4 2-Tc::Mu-Kn::Tn7 (Tp^R^ Str^R^)	Simon et al., 1983
Top10 F'	F'citation{*lacI*^*q*^ Tn10 (Tet^R^)} *mcrA* Δ(*mrr-hsd RMS-mcrBC*) *ϕ80lacZ ΔM15 Δ lacX74 deoR recA1 araD139 Δ99ara-leu* 7697	Invitrogen
**PLASMIDS**		
pDrive	cloning vector, Amp^R^, Km^R^	Qiagen
pEP185.2	suicide vector, Cm^R^	Kinder et al., 1993
pFUSE	suicide vector, derivative of pEP185.2 with promoterless lacZYA genes, Cm^R^	Baumler et al., 1996
ER3	pEP185.2 with XbaI/SmaI fragment (249 bp) of *rovA*, Cm^R^	This work
pFR1	pFUSE with XbaI/SmaI fragment (412 bp) of *rovA*, Cm^R^	This work
pET28a	expression vector with 6His-tag coding sequence, Km^R^	Novagen
ETR1	pET28a with 432 bp fragment representing the entire *rovA* coding sequence, Km^R^	This work
pETRlac	pETR1 containing BglII/XbaI DNA fragment with *lac* promoter, Km^R^	This work

To monitor the influence of pH of the growth medium, an overnight culture was grown and variation of the pH was achieved by buffering the growth medium with MOPS [3-(N-morpholino) propanesulfonic acid—pH 7.0, 7.5, 8.0, 8.5], MES [2-(N-morpholino)ethanesulfonic acid—pH 5.5, 6.0, 6.5], or homoPIPES [homopiperazine-*N*,*N*'-bis(2-ethanesulfonic acid)—pH 5.0] at 0.1 M. Where appropriate, antibiotics were added to media at the following concentrations: chloramphenicol, 25 μg ml^−1^; kanamycin, 50 μg ml^−1^; nalidixic acid, 20 μg ml^−1^; tetracycline 12.5 μg ml^−1^.

### DNA techniques

DNA manipulations, such as restriction digestion, ligation, transformation, and conjugation were performed using standard protocols (Sambrook et al., 1989). Plasmid and chromosomal DNA were purified using Invitrogen kits. DNA fragments were amplified by PCR using *Taq* DNA polymerase (Invitrogen) and oligonucleotide primers. PCR products were purified directly using the PureLink PCR purification kit (Invitrogen), or following agarose gel electrophoresis, with the PureLink Gel extraction kit (Invitrogen).

### β-galactosidase assays

β-Galactosidase activities were assayed by the method of Miller (1972) with ONPG (o-nitrophenyl-β-galactopyranoside) as a substrate. Routinely, triplicate cultures were grown for each assay and the assays were repeated at least twice.

### Construction of *Y. enterocolitica rovA* insertion mutant

Gene inactivation in *Y. enterocolitica* strains was performed by plasmid insertion through homologous recombination using the conjugative suicide vector pEP185.2 (Kinder et al., 1993). A 249-bp intragenic fragment of *rovA* was amplified using the primers rovA1 (5′-TGTCTAGAGGTATGGCAGGACAAGGTGT-3′) and rovA249 (5′-TGCCCGGGAAGCCAGAGATCGCAATGAT-3′). The DNA fragment was subcloned into the pDrive cloning vector (Qiagen), then excised with restriction enzymes XbaI and SmaI and subsequently ligated with XbaI/SmaI-digested pEP185.2. The resulting construct, pER3, was transferred from *E. coli* S17-1 λ*pir* to *Y. enterocolitica* strains AR4 and/or Ye9N by biparental conjugation. Strains harboring plasmids integrated into the chromosome were recovered by selecting for Cm^r^. The insertion mutant strains obtained by this strategy were designated AS3 (*rovA*) and AC1 (*ompR, rovA*). Correct integration at the *rovA* locus was confirmed by PCR with one primer located upstream of the homologous region used for recombination and the other within the chloramphenicol resistance cassette of the suicide vector (data not shown).

### Construction of pETR1 and pETRlac plasmids

To create pETR1, a 432-bp fragment representing the entire *rovA* coding sequence was amplified using primers RovApET1 (5′-CATGCCATGGATGGAATCGACATTAGGATCTGA-3′) and RovApET2 (5′-CCGCTCGAGCTTACTTTGTAGTTGAATAATGTTTCTCTC-3′). The PCR product was digested with XhoI and NcoI and ligated with XhoI/NcoI-cleaved vector pET28a. The resulting vector expresses RovA fusion protein with an amino-terminal His_6_ extension.

To obtain pETRlac a 423-bp DNA fragment containing the *lac* promoter was amplified by PCR with primers Lac1B (5′-TGAGATCTTATGGAAAAACGCCAGCAAC-3′) and Lac423X (5′-TGTCTAGATGGCGTAATCATGGTCATAGC-3′) using pBluescript SK II (+) as a template. The PCR product was digested with BglII and XbaI and cloned into the BglII/XbaI site of pETR1. The resulting vector expresses the RovA protein under the transcriptional control of the *lac* promoter instead of the excised T7 promoter. To complement the *rovA* mutation, the pETRlac plasmid was introduced into *Y. enterocolitica* strain AS3 by electroporation. Strains harboring the plasmid were recovered by selecting for Km^r^.

### Semi-quantitative reverse transcription (RT)-PCR gene expression analysis

Total RNA was extracted from strains of *Y. enterocolitica* grown under different conditions using a GF-1 Nucleic Acid Extraction Kit (Vivantis). This RNA was treated with RNase-free DNase I (Invitrogen) and quantified by spectrophotometry (absorbance at 260 nm). cDNA was synthesized using the SuperScript III First-Strand Synthesis System for RT-PCR (Invitrogen). To exclude the possibility of DNA contamination, minus-RT controls (without the reverse transcriptase) were prepared from RNA samples. The cDNA concentration for expression analysis was normalized using PCR with primers amplifying a 211-bp fragment of the constitutively expressed *Y. enterocolitica* 16S rRNA gene (forward primer F16SRT 5′-TACGCATTTCACCGCTACAC-3′; reverse primer R16SRT 5′-CAGAAGAAGCACCGGCTAAC-3′). Primer pairs were designed to amplify 384-bp *inv* fragment (FinvRT 5′-ACCCTGTACCCAATACCGAAG-3′ and RinvRT 5′-CTCGATCAGCGCAGTAAAATC-3′) and 239 bp *ompR* fragment (FompR250 5′-GCTCTAGAGCCAAGGGTGAAGAAGTTGA-3′ and RompR489 5′-TCCCCCGGGGCTGGTCAGTGGCATAGGTT-3′). The primers were used with different cDNA preparations in PCRs to semi-quantitatively compare the expression level of these genes. The number of cycles used varied according to the abundance of the various mRNAs to ensure that the comparisons were performed in the linear range of amplification: 10–16 cycles for the 16S rRNA gene, 23–28 for *inv* and 23–25 for *ompR*. The separately amplified products of 16S rRNA gene and the analyzed genes (*inv* or *ompR*) were mixed and loaded together onto 2% TAE agarose gels, separated by electrophoresis and stained with ethidium bromide. Band intensities were quantified using ImageMaster VDS (Amersham Pharmacia Biotech) with Quantity One software (Bio-Rad). RT-PCR values are presented as a ratio of the specified gene signal divided by the 16S rRNA signal. Statistical significance was calculated using ANOVA and Tukey's *post-hoc* multiple mean comparison test. Tukey's test compares each RT-PCR signals mean in a pairwise manner. Statistical significance was accepted at *P* < 0.05.

### Western immunoblotting

The expression of OmpR protein in *Y. enterocolitica* cells was evaluated by Western blot analysis. Equal numbers of bacterial cells, grown under different conditions, were resuspended in 10 mM phosphate buffer (pH 8.0) containing 1 mM phenylmethylsulfonyl fluoride (PMSF) and sonicated. After centrifugation of the cell extracts (15,500× g, 30 min, 4°C) the supernatant fractions were collected and the total protein in each sample was quantified (Bio-Rad Protein Assay). Equal amounts (8 μ g) of total protein from each sample were mixed with 2×sodium dodecyl sulfate (SDS)-electrophoresis loading buffer and boiled for 5 min. Electrophoresis of samples was carried out on SDS-urea polyacrylamide gels (12% polyacrylamide, 6 M urea). The gels were then blotted onto Immun-blot PVDF membrane (Bio-Rad) using a semi-dry transfer unit (Hoeffer Scientific Instruments) for 1 h at 50 V following the procedure of Towbin et al. (1979). The OmpR protein was detected on the blots by probing with a 1:5000 dilution of a rabbit polyclonal antibody raised against purified OmpR-His_6_ (Brzostek et al., 2007). Then, secondary alkaline phosphatase-conjugated goat anti-rabbit antibody was applied (1:1000) (Roche). Immunocomplexes were visualized using the chromogenic substrate nitroblue tetrazolium/5-bromo-4-chloro-3-indolylphosphate (NBT/BCIP; Roche).

### Construction of A *rovA*::*lacZYA* chromosomal transcriptional fusion

To obtain a chromosomal *rovA::lacZYA* transcriptional fusion, a 412-bp fragment of DNA encompassing a 3′- end of the *rovA* gene, was amplified by PCR using the oligonucleotides rovA1X (5′-TGTCTAGATGATTTAGCACGATTAGTTCG-3′) and rovA432S (5′-TGCCCGGGTTACTTACTTTGTAGTTGAATAATG-3′), and the product was cloned into the cloning vector pDrive (Qiagen). The XbaI/SmaI *rovA* fragment was then subcloned into XbaI/SmaI digested pFUSE, a suicide vector that carries the promoterless *lacZYA* operon (Baumler et al., 1996). The resulting construct pFR1 was propagated in *E. coli* strain S17-1 λ*pir* and transferred to *Y. enterocolitica* strains Ye9N and AR4 by biparental conjugation. Transconjugants YeR2 and ARR8 were selected on LB plates supplemented with Nal and Cm in the case of recipient strain Ye9N, or Nal, Cm and Km for AR4. The recombination of the plasmid into the chromosome yielded strains, which carry a complete wild-type copy of the *rovA* gene. Correct integration at the *rovA* locus was confirmed by PCR and DNA sequencing (data not shown). PCRs were carried out using primers rovA01 (5′-TGAGAGCTCGACTTTGCCATCACGAGTCC-3′) and placZ (5′-AGTCTCAATCTGCACTACAA-3′), which amplify a region starting before the *rovA* gene and including part of the *lacZ* sequence present in pFUSE.

The functionality of the *rovA* promoter driving *lacZYA* expression in the selected transconjugant strains was confirmed by the production of a blue color following growth at 25°C on LB agar plates supplemented with 5-bromo-4-chloro-3-indolyl-β-D-galactopyranoside (20 μg ml^−1^).

The β-galactosidase activity of strains YeR2 and ARR8 grown under different temperature and pH conditions was measured by monitoring the degradation of *o*-nitrophenyl-β-D-galactoside into *o*-nitrophenol, which absorbs at 420 nm.

### Overproduction and purification of OmpR-His_6_ and RovA-His_6_

The *ompR* structural gene was cloned in the expression vector pQE30 and an N-terminal His-tagged OmpR hybrid protein (OmpR-His_6_) was synthesized in *E. coli* M-15 and purified as described previously (Brzostek et al., 2007). Plasmid pETR1 carrying the entire *rovA* coding sequence under the control of the T7 promoter (see above) was used to transform *E. coli* BL21 (DE3). Expression and purification of the C-terminal His-tagged RovA hybrid protein (RovA-His_6_) was performed with Ni-NTA resin (Qiagen) according to the manufacturer's standard protocol. Briefly, *E. coli* BL21 (DE3) carrying plasmid pETR1 was grown to mid-logarithmic phase and induced with IPTG (1 mM) for 4 h at 30°C. The cells were then pelleted by centrifugation, resuspended in 50 mM phosphate buffer (pH 8.0) containing 300 mM NaCl, 55 μM PMSF, 5 mM imidazole and 10 mM 2-mercaptoethanol, and disrupted by sonication. The sample was centrifuged and the supernatant passed through a Ni-NTA agarose column. The RovA-His_6_ protein was eluted from the column in 50 mM phosphate buffer (pH 8.0) containing 300 mM NaCl and 125 mM imidazole, and then dialyzed at 4°C in 10 mM Tris-HCl (pH 7.5) buffer containing 5 mM 2-mercaptoethanol, 10 mM NaCl and 5% glycerol. Aliquots of purified RovA-His_6_ and OmpR-His_6_ hybrid proteins were stored at −70°C. Protein concentrations were determined using a Pierce BCA Protein assay kit with bovine serum albumin as the standard.

### Electrophoretic mobility shift assays (EMSAs)

A 553-bp fragment of the *inv* promotor region (−328 to +225 bp) encompassing the OmpR and RovA binding sites was obtained by PCR with primers GSinvF (5′-ATGACATCGCCATCACACTG-3′) and GSinvR (5′-TTTTGCTGTGAGAACCCATAA-3′). The purified fragment (~ 20 ng in 15 μl) was incubated for 30 min at room temperature with RovA-His_6_ or OmpR-His_6_ in the presence of the binding buffer (40 mM Tris-HCl pH 8.0, 100 mM KCl, 10 mM MgCl_2_, 5% glycerol) and the reactions were analyzed by electrophoresis in 6% native polyacrylamide gels (29:1 acrylamide/bis acrylamide) in 0.5 × TBE buffer. In some binding reactions the OmpR-His_6_ used was phosphorylated by 30 min treatment with 20 mM acetyl phosphate (Sigma). The DNA bands were visualized by silver staining using reagents in a kit, according to the manufacturer's protocol (Kucharczyk) or with ethidium bromide. Competitive EMSAs were performed by incubating the DNA first with RovA-His_6_ protein followed by the addition of increasing amounts of OmpR-P-His_6,_ or the inverse, where RovA-His_6_ was added to reactions in which the DNA had first been incubated with OmpR-P-His_6_. As negative controls to confirm binding specificity, a 307-bp fragment of 16S rDNA of *Y. enterocolitica* Ye9 or a 354-bp fragment of the *ngoA302V* gene from *Neisserria gonorrhoeae* FA1090 were included in the binding reactions. The PCR fragment of 16S rDNA was generated by using primer 16SR1 5′-ATTCCGATTAACGCTTGCAC-3′ and 16SR304 5′-GTGGGGTAATGGCTCACCTA-3′, the PCR *ngoA302V* fragment by primer VsrA1 5′-ACGCGTCGACCATGGATAAATTAACC-3′ and VsrA354 5′-GCCAACACAAGAGCGGGTTTCGTCC-3′.

### Sample preparation and protein identification by the liquid chromatography-coupled tandem mass spectrometry (LC-MS/MS)

For the LC-MS/MS analysis, the DNA-protein complexes from the EMSA examining the competition between RovA (which was added first) and OmpR were separated in 6% native polyacrylamide gel and stained with ethidium bromide. The slice of gel containing the shifted band from EMSA was excised with a clean scalpel. *Prior* to the LC-MS/MS analysis excised gel slice was subjected to the standard procedure of in-gel trypsin digestion, during which proteins were reduced with 100 mM DTT for 30 min at 56°C, alkylated with iodoacetamide in darkness for 45 min at room temperature, and digested overnight with 10 ng/ml trypsin. Peptides were eluted from gel with the water solution of 0.1% formic acid and 2% acetonitrile. Separation of peptides with high pressure liquid chromatography (nano-HPLC RP-18 column, 75 μM id, Waters, Milford MA) and subsequent tandem mass spectrometry analysis (ESI-LTQ-FTICR, Thermo Electron Corp., San Jose, CA) was performed at the Mass Spectrometry Laboratory of Institute of Biochemistry and Biophysics, Polish Academy of Sciences, Warsaw, Poland. After preprocessing of the raw data with Mascot Distiller software (version 2.2.1, Matrix Science, London, UK), obtained peak lists were used to search the non-redundant protein database of the National Center for Biotechnology Information (NCBI) (10391716 sequences; 3545023166 residues) using the MASCOT search engine (version 2.2.03, 8-processors on-site license) (Matrix Science, London, UK).

## Results

### Effect of temperature and pH conditions on *inv* transcription in Ye9 strain

**Figure 1:**
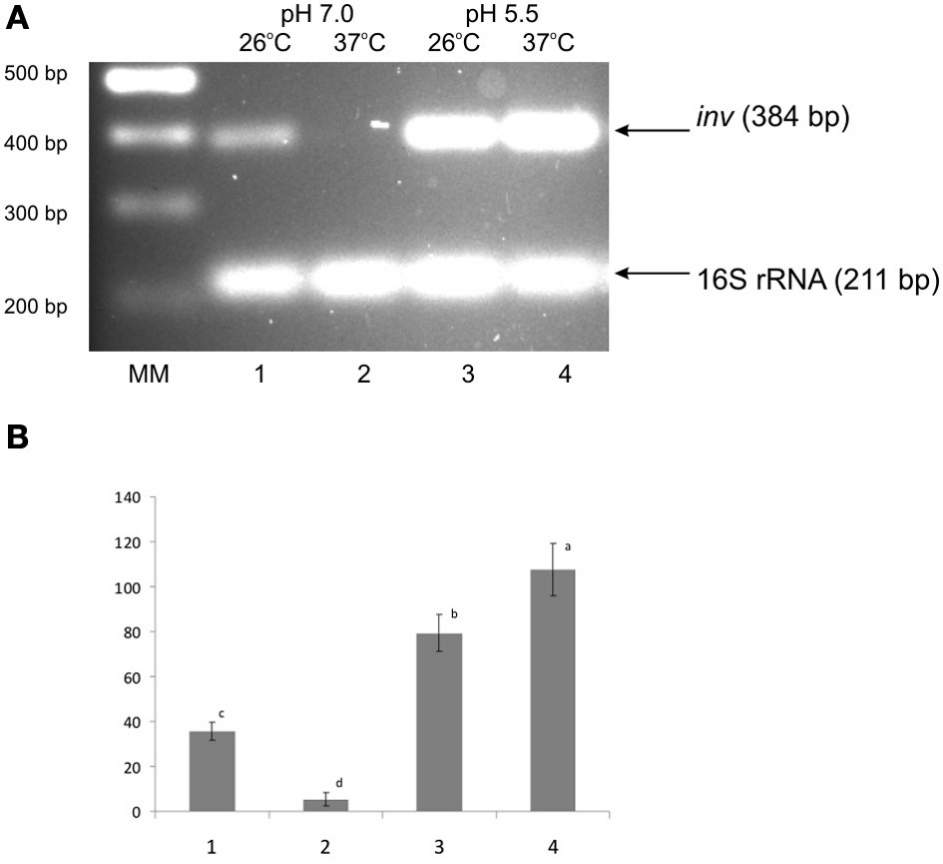
**Effect of pH and temperature on *inv* transcription in the wild-type strain Ye9.** Cells were grown to stationary phase at 25°C or 37°C in LB medium buffered to pH 7.0 or 5.5. Total RNA was extracted and used in sqRT-PCR to assess *inv* mRNA levels. PCRs for *inv* and 16S rRNA were carried out for 23 cycles and 10 cycles, respectively. **(A**) Lanes: MM—DNA molecular mass marker (100 bp ladder); lane 1–25°C, pH 7.0; lane 2—37°C, pH 7.0; lane 3—25°C, pH 5.5; lane 4—37°C, pH 5.5. **(B)** The densities of *inv* bands relative to those of the 16S rRNA bands on the gel in part A. Values are means ± SD, *n* = 2–3; a, b, c, d—results of Tukey *post-hoc* multiple mean comparison test. Means without a common letter differ significantly (*p* < 0.05).

Early observations indicated that at neutral pH the expression of *inv* in *Y. enterocolitica* 8081 strain (serotype O8) is activated at 26°C and strongly repressed at 37°C. In contrast, cells grown at 37°C at pH 5.5 exhibit the level of *inv* expression comparable to those at 26°C (Pepe et al., 1994). This low pH-dependent up-regulation of *inv* expression at 37°C has not been described for *Y. pseudotuberculosis* (Nagel et al., 2001). Recently, high and constitutive expression of *inv* at neutral pH has been described for *Y. enterocolitica* serotype O3 strains grown at 25°C and 37°C (Uliczka et al., 2011). To investigate whether the pH-dependent regulation of *inv* occurs in Ye9 strain, RT-PCR analyses were carried out to study *inv* transcription. Figure 1 shows that the level of *inv* mRNA in cells grown at 37°C and pH 7.0 was dramatically reduced, i.e., the *inv* transcript was not or barely visible. Furthermore, *inv* transcription was elevated at both temperatures when the pH was 5.5. These data suggest that in *Y. enterocolitica* Ye9 strain (O9 serotype), the molecular control of *inv* transcription in response to changes in pH differs from that described for other *Y. enterocolitica* serotypes/strains.

### The expression pattern of *OmpR* in response to different temperature and pH conditions

**Figure 2:**
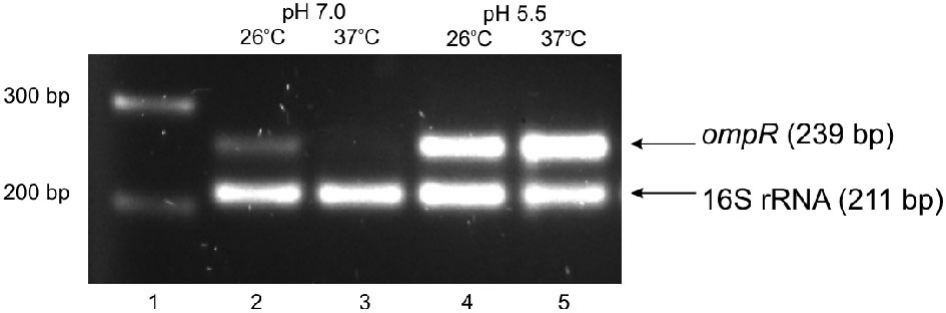
**Effect of pH and temperature on *ompR* transcription in the wild-type strain Ye9.** Cells were grown to early stationary phase at 25°C or 37°C in LB medium buffered to pH 7.0 or 5.5. Total RNA was extracted and used in sqRT-PCR to assess *ompR* mRNA levels. PCRs for *ompR* and 16S rRNA were carried out for 25 cycles and 10 cycles, respectively. Lanes: 1—DNA molecular mass marker (100 bp ladder); lane 2—25°C, pH 7.0; lane 3—37°C, pH 7.0; lane 4—25°C, pH 5.5; lane 5—37°C, pH 5.5.

**Figure 3:**
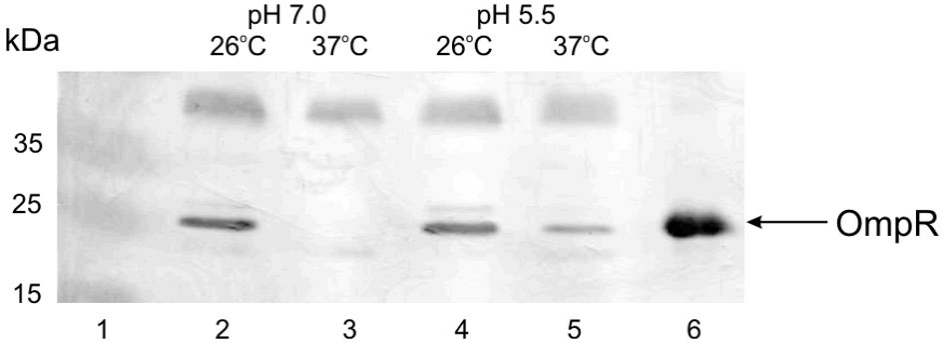
**OmpR protein levels present in Ye9 cells grown under different conditions.** Cytoplasmic extracts of cells grown to early stationary phase in buffered LB medium were Western blotted and probed with anti-OmpR antibody. Lanes: 1—PageRuler Prestained Protein Ladder Plus (Fermentas); lane 2—25°C, pH 7.0; lane 3—37°C, pH 7.0; lane 4—25°C, pH 5.5; lane 5—37°C, pH 5.5; lane 6—OmpR-His_6_ protein (1.5 μg).

The regulation of virulence genes by environmental cues is achieved by alterations in the level or activity of regulatory proteins. It has been shown previously that phosphorylation of OmpR activates this regulatory protein (McCleary and Stock, 1994; Lan and Igo, 1998). Just as the activation/phosphorylation of OmpR might influence *inv* transcription, so too might the expression of *ompR*. To study whether *ompR* expression responds to changes in temperature and pH, the levels of the *ompR* transcript in wild-type Ye9 cells were analyzed by sqRT-PCR. We examined *ompR* transcript abundance in strain Ye9 grown to early stationary phase at 25°C and 37°C in buffered LB medium at pH 7.0 or 5.5 (Figure 2). The *ompR* mRNA level at neutral pH 7.0 was markedly lower at 37°C compared with 25°C, which indicated that *ompR* transcription is temperature-dependent. Moreover, we found an increase in the level of the *ompR* transcript at pH 5.5 at both temperatures, although the degree of response to low pH was slightly higher at 37°C than at 25°C. To determine whether the differences in the transcription of *ompR* in strain Ye9 were reflected in the level of OmpR protein, Western blot analysis was performed using a polyclonal antibody raised against purified OmpR (Figure 3). Immunoblotting of cytoplasmic proteins of strain Ye9 grown at pH 7.0 showed no trace of OmpR protein at 37°C, whereas in the extract from cells at 25°C, a clear immunoreactive band was detected. In cells propagated at pH 5.5, increased OmpR protein levels were observed at both temperatures. These data revealed that levels of OmpR protein essentially correlate with the amount of *ompR* transcript in cells grown at different temperatures (25 and 37°C) and pH (pH 7.0 and 5.5).

### Effect of growth conditions and OmpR activity on *rovA* promoter function

**Figure 4:**
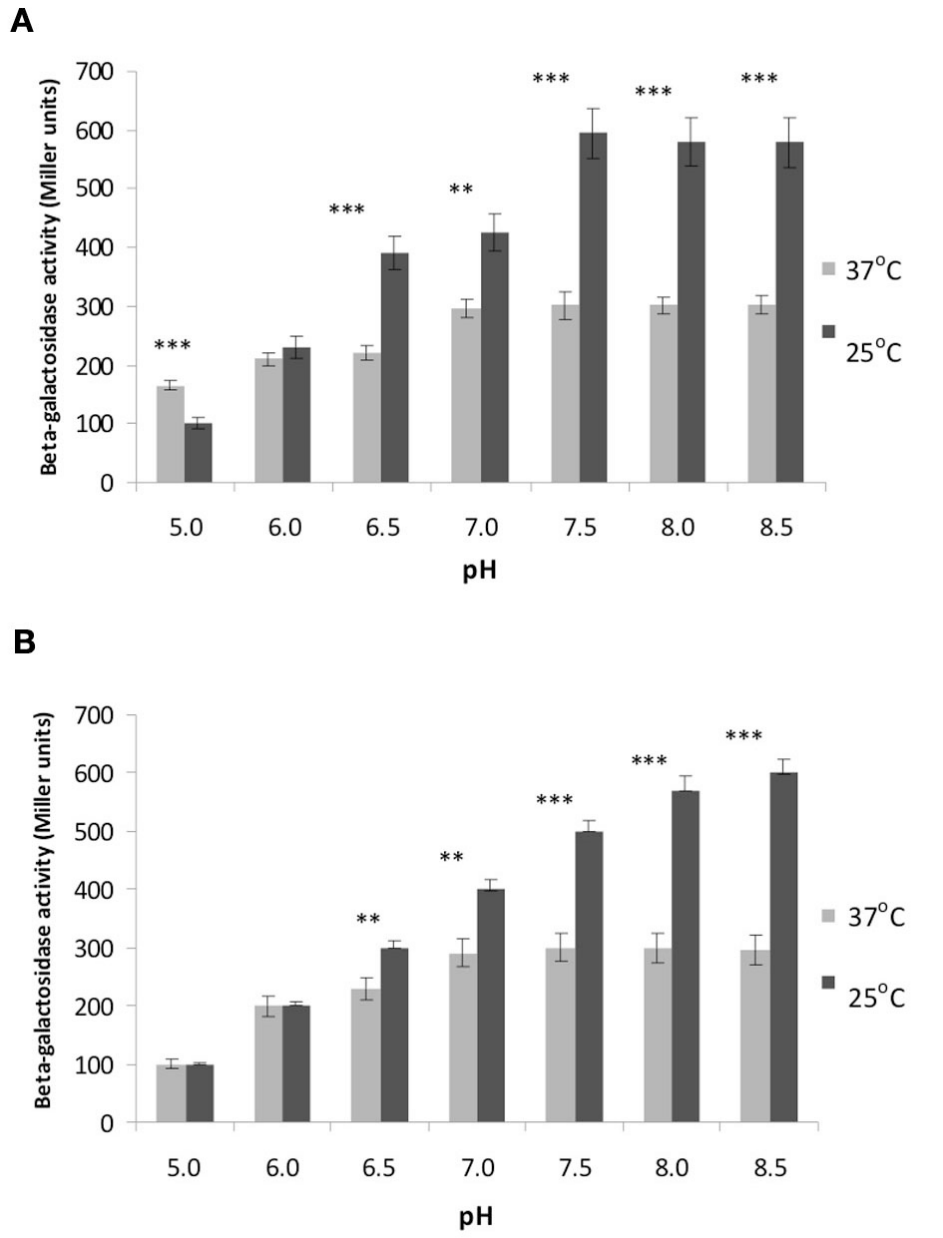
**Effect of OmpR and different temperature and pH conditions on *rovA* expression determined using a *rovA*::*lacZYA* operon fusion.** β-galactosidase activities were measured in strain YeR2 (OmpR^+^) and in the isogenic mutant strain ARR8 (OmpR^−^) grown to early stationary phase in LB medium at different pH at 25°C **(A)** or 37°C **(B)**. The data presented are the means of three-independent experiments ± SD. Statistical significance was calculated using Student's *t*-test. ^**^p < 0.01; ^***^p < 0.001.

Our previous *in vitro* studies showed that OmpR from strain Ye9 is able to bind specifically to the *inv* promoter region leading to the repression of *inv* transcription. As well as having a possible direct effect on *inv* expression, OmpR may also influence *rovA* expression. To determine whether the loss of OmpR alters *rovA* expression, a *rovA*::*lacZYA* chromosomal transcriptional fusion was created in *Y. enterocolitica* strain Ye9N and AR4 (*ompR* mutant) via homologous recombination, yielding strains YeR2 (OmpR^+^) and ARR8 (OmpR^−^), respectively. The β-galactosidase activity was then measured in both strains grown under different pH and temperature conditions (Figures 4A,B). We found that pH significantly influences the β-galactosidase activity at different temperatures (ANOVA: for YeR2, 25°C *F*_(6.14)_= 34.510, *p* < 0.001; for YeR2, 37°C *F*_(6.14)_ = 14.008, *p* < 0.001; for ARR8, 25°C *F*_(6.14)_ = 130.551, *p* < 0.001; for ARR8, 37°C *F*_(6.14)_ = 12.673, *p* < 0.001).

The optimal pH for *rovA* expression in YeR2 (OmpR^+^) and ARR8 (OmpR^−^) was found to be 7.5 at both temperatures and raising the pH to 8.5 did not lead to changed *rovA* promoter activity. However, we observed that the activity of *rovA* promoter, measured by assaying for β-galactosidase activity, was about 2-fold lower in YeR2 and ARR8 cells grown at pH 7.5 at 37°C compared with 25°C (*p* < 0.001). Moreover, a shift to acidic pH values at 25°C resulted in a significant fall in *rovA* activity with the greatest decrease of ~6-fold occurring when the pH was reduced to 5.0 (*p* < 0.001). Analysis of *rovA* expression in YeR2 and ARR8 cells grown at 37°C under different pH conditions (at pH values below 7.5) demonstrated also a significant reduction in *rovA* promoter activity. However, a shift from pH 7.5 to 5.0 resulted only in a ~2-fold decrease for YeR2 cells.

In summary, our data demonstrated temperature-dependent expression of *rovA*, namely reduced *rovA* expression at 37°C compared with that at 26°C, confirming the results previously obtained for *Y. enterocolitica* serotype O8 and *Y. pseudotuberculosis* (Heroven et al., 2004; Ellison and Miller, 2006b). In addition, no significant differences (*p* > 0.05) in the activity of *rovA* measured under different pH and temperature conditions were observed in cells with and without OmpR, indicating that OmpR does not influence *rovA* expression.

In contrast, at pH 5.0 at 37°C significant differences (*p* < 0.05) in the activity of *rovA* promoter were observed in cells with and without OmpR, indicating that OmpR might influence *rovA* expression under these particular conditions.

### OmpR- and RovA-dependent *inv* regulation in *Y. enterocolitica*

**Figure 5:**
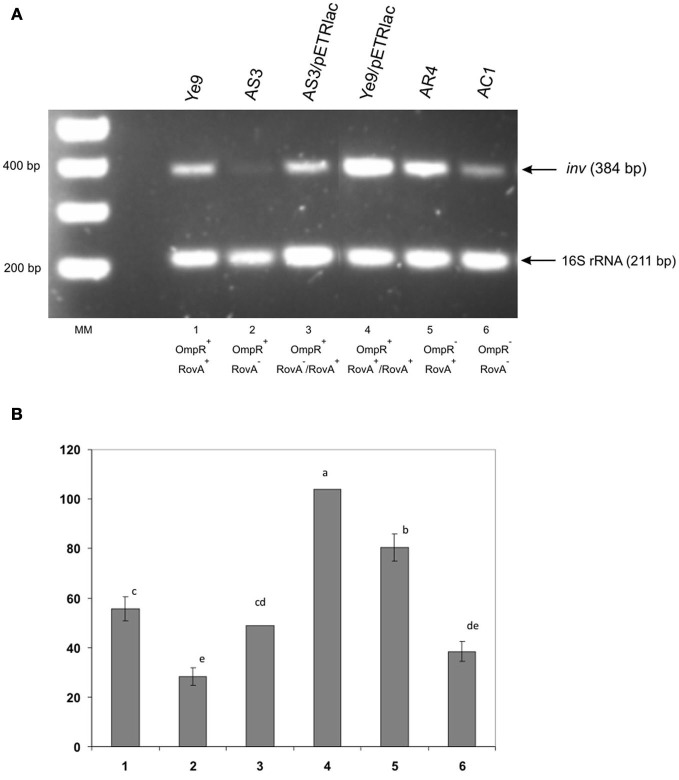
**Influence of OmpR and RovA proteins on *inv* transcription in wild-type *Y. enterocolitica*, *rovA*, *ompR* and *rovAompR* mutants, and complemented strains.** Cells were grown to early stationary phase at 25°C in LB medium (pH 7.0). Total RNA was extracted and used in sqRT-PCR to assess *inv* mRNA levels. PCRs for *inv* and 16S rRNA were carried out for 28 cycles and 16 cycles, respectively. The PCR reactions were mixed before loading onto the gel. **(A)** Lanes: MM—DNA molecular mass marker (100 bp ladder); 1—Ye9 (WT); 2—AS3 (*rovA* mutant); 3—AS3/pETRlac; 4—Ye9/pETRlac; 5—AR4 (*ompR* mutant); 6—AC1 (*ompRrovA* mutant). **(B)** The densities of *inv* bands relative to those of the 16S rRNA bands on the gel in part A. RT-PCR signals were averaged from 3 replicates (lanes 1, 2, 5, 6). Values are means ± SD; a, b, c, d, e—results of Tukey *post-hoc* multiple mean comparison test. Means without a common letter differ significantly (*p* < 0.05).

The expression of *inv* in *Y. enterocolitica* is known to be positively regulated by RovA protein acting mainly as an anti-repressor of the H-NS/YmoA complex. To determine whether the inhibitory effect of OmpR correlates with RovA activity, a *rovA* null mutation was introduced into strains Ye9 and AR4 (*ompR* mutant) by insertional mutagenesis using the plasmid pEP185.2. The engineered *Y. enterocolitica* (*rovA*::pEP185.2) mutants were named AS3 and AC1, respectively. To perform complementation analyses, plasmid pETRlac (carrying the cloned *rovA* gene) was introduced *in trans* to the strain AS3. The effect of overproduction of RovA in *Y. enterocolitica* cells was monitored in wild-type strain Ye9 carrying plasmid pETRlac. The level of *inv* transcription was examined in cells grown in LB medium at neutral pH and moderate temperature—conditions known to produce high level *inv* expression. SqRT-PCR was used to measure changes in the mRNA level in *rovA*, *ompR*, and *rovA ompR* mutants (Figure 5). As anticipated, higher levels (increase by 35%) of *inv* mRNA were observed in the *ompR* mutant AR4 compared with the wild-type strain Ye9 indicating the negative role played by OmpR. (Figure 5, lane 5 vs. 1). In contrast, the level of the *inv* transcript was reduced 2-fold in the *rovA* mutant background of strain AS3 (Figure 5, lane 2 vs. 1). The positive effect of RovA on *inv* transcription was confirmed by complementation analysis where the plasmid pETRlac was introduced *in trans* to the *rovA* mutant. RovA protein produced by pETRlac restored the activity of the *inv* promoter almost to the wild-type level (Figure 5, lane 3 vs. 2). The increased level of RovA expressed from pETRlac also led to much (nearly 2-fold) higher *inv* expression in wild-type cells (Figure 5, lane 4 vs. 1). Moreover, when the *inv* transcription was relieved from the negative effect of the OmpR protein in the *rovA* mutant background of strain AC1 (*ompR, rovA*), the level of *inv* mRNA decreased over 2-fold compared with that observed in *ompR* mutant AR4 with active RovA (Figure 5, lane 6 vs. 5). These data indicated that removal of OmpR leads to higher *inv* expression level only in the presence of RovA. Conversely, when RovA is absent, the inhibition of *inv* transcription can be seen regardless of the presence of OmpR and probably results from the activity of the H-NS/YmoA repression complex.

### Interaction of OmpR and RovA with the *inv* promoter region *in vitro*

**Figure 6:**
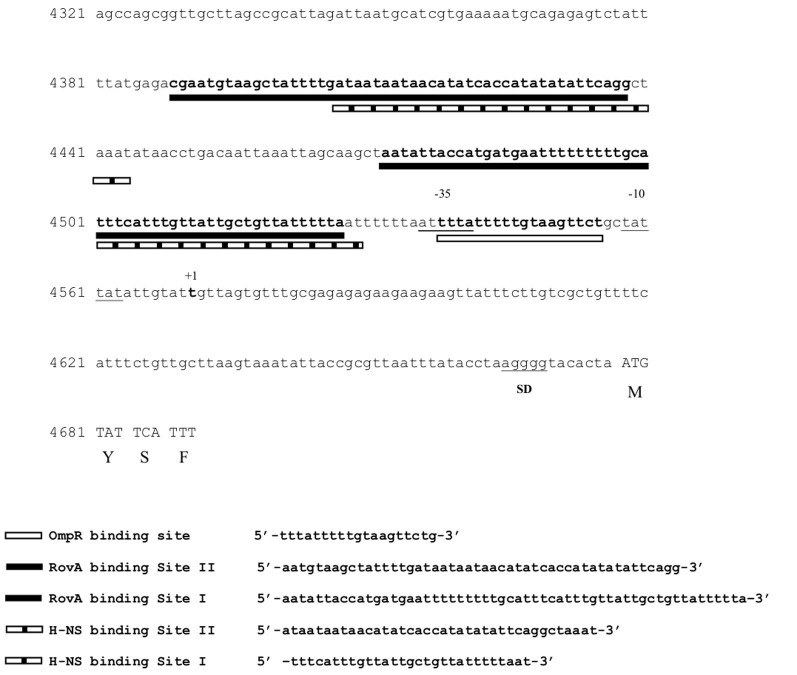
**OmpR, RovA and H-NS binding sites in the promoter region of *inv* in *Y. enterocolitica*.** Two RovA and H-NS binding sites (I and II), (Ellison and Miller, 2006b) and the putative OmpR binding site (Brzostek et al., 2007) are underlined. The transcriptional start site of the *inv* promoter (+1), ATG start codon and Shine-Dalgarno (SD) sequences are indicated.

A detailed characterization of RovA in *Y. enterocolitica* and *Y. pseudotuberculosis* showed that this regulator positively regulates *inv* expression by acting mainly as a derepressor that competes with H-NS for binding sites within the *inv* promoter (Heroven et al., 2004; Ellison and Miller, 2006b). Two RovA binding sites, previously recognized in *Y. enterocolitica* O8 *inv* promoter, are located between −177 and −38 bp relative to the transcriptional start site and overlap with two H-NS binding sites (Figure 6). In addition, the examination of *inv* promoter sequence indicated that RovA binding sites and the putative OmpR binding site (between −15 and −33 bp), predicted by *in silico* analysis and confirmed by *in vitro* band-shift assays (Brzostek et al., 2007), do not overlap. Thus, these regulators may bind independently to the *inv* promoter, although the binding of one of these proteins to DNA may influence the interaction of the other. To gain further insight into the interactions of OmpR and RovA with the *inv* promoter, electrophoretic mobility shift assays (EMSAs) were performed.

**Figure 7:**
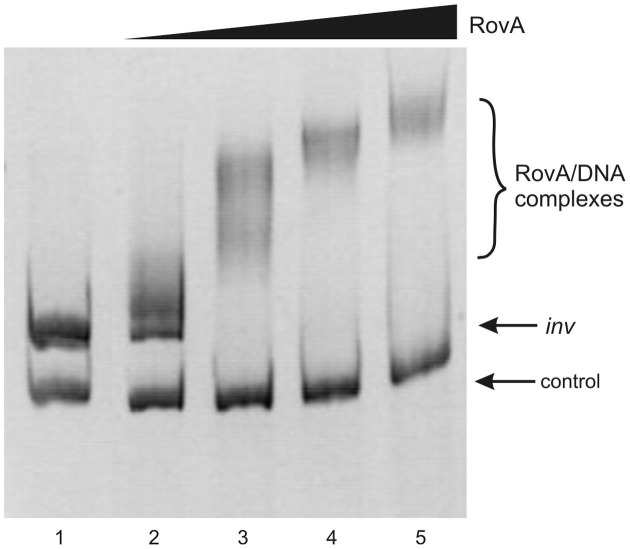
**Interaction of purified RovA from Ye9 strain with the *inv* promoter region examined in EMSA.** EMSA showing the binding of increasing quantities of RovA-His_6_ to the *inv* promoter region, using a 553-bp *inv* DNA fragment (−328 to +225) encompassing the RovA binding sites. The amount of RovA added was 0, 0.125, 0.25, 0.5, and 1.0 μg (lanes 1–5). A 300-bp fragment of the *ngoA302V* gene from *Neisserria gonorrhoeae* FA1090 was used as a negative control. DNA-protein complexes were separated by electrophoresis in 6% native polyacrylamide gels and silver stained.

**Figure 8:**
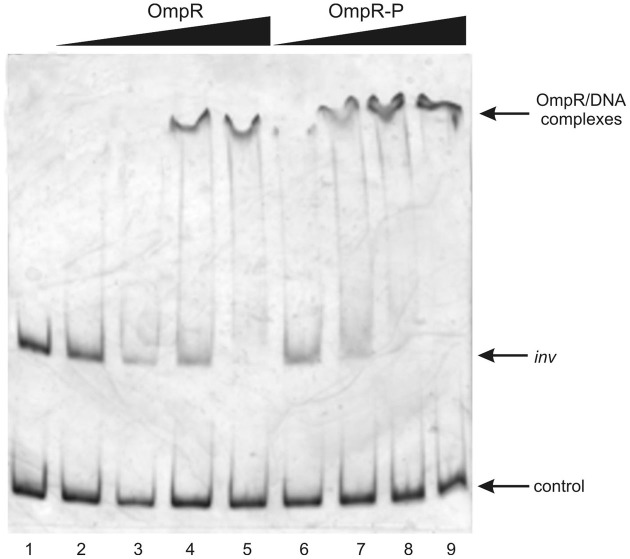
**Interaction of purified OmpR with the *inv* promoter region examined in EMSA.** EMSA showing the binding of increasing quantities of non-phosphorylated (OmpR; 0.1, 0.2, 0.3, 0.4 μg—lanes 2–5) or phosphorylated (OmpR-P; 0.1, 0.2, 0.3, 0.4 μg—lanes 6–9) OmpR-His_6_ protein to the *inv* promoter region. A 553-bp *inv* DNA fragment (−328 to +225) encompassing the OmpR and RovA binding sites was used. A 307-bp fragment of 16S rDNA of *Y. enterocolitica* was used as a negative control. Lane 1- *inv* promoter fragment and control DNA incubated without proteins. DNA-protein complexes were separated by electrophoresis in 6% native polyacrylamide gels and silver stained.

To determine whether both RovA and OmpR bind specifically to the *inv* promoter region, a PCR-amplified 553-bp fragment comprising the binding sites of both these proteins was used in band-shift assays. Recombinant OmpR-His_6_ and RovA-His_6_ proteins were expressed in *E. coli* and purified to homogeneity by affinity chromatography using Ni-NTA agarose. The purity of the RovA (18 kDa) and OmpR (27 kDa) hybrid proteins was verified by electrophoresis on 12% SDS-polyacrylamide gels (data not shown). Different amounts of the purified proteins were incubated with the *inv* promoter fragment and these binding reactions were analyzed by electrophoresis in 6% native polyacrylamide gels. The results presented in Figure 7 demonstrate that RovA from *Y. enterocolitica* Ye9 binds to the *inv* promoter fragment to form DNA-protein complexes. An apparent stepwise shifting of the *inv* fragment with increasing amounts of RovA (0.125–1.0 μg) was observed, suggesting the presence of more than one RovA binding site in the *inv* promoter region. The interaction of RovA with the *inv* promoter appears to be specific, since at the concentration required for binding of the *inv* promoter fragment, this protein did not bind the 300-bp control fragment derived from the *ngoA302V* gene of *Neisserria gonorrhoeae* FA1090. The EMSA using OmpR demonstrated that the interaction of this protein with the *inv* promoter fragment produced one visible nucleoprotein band irrespective of the amount of OmpR added to the binding reaction (0.1–0.4 μg) (Figure 8). Furthermore, differences in the mobility of the shifted DNA-protein complex were observed when non-phosphorylated and phosphorylated (by acetyl-P) forms of OmpR were used (Figure 8, lanes 2–5 vs. 6–9). In addition, the minimum amount of phosphorylated OmpR that was able to bind the *inv* promoter fragment was approximately 3-fold lower than that for the non-phosphorylated OmpR. No mobility shift of the 16S rDNA control fragment was detected under any of the conditions tested.

**Figure 9:**
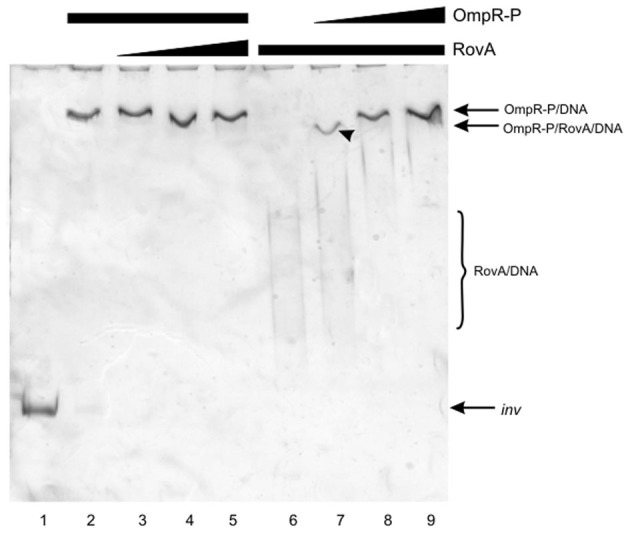
**Competition for binding the *inv* promoter fragment between OmpR and RovA proteins.** EMSAs examining competition for binding the *inv* promoter fragment between OmpR-P, which was added first (0.3 μg—lanes 2–5), and RovA (0.05, 0.125, 0.25 μg, lanes 3–5 respectively); and between RovA, which was added first (0.25 μg, lanes 6–9), and OmpR-P (0.05, 0.15, 0.3 μg—lanes 7–9). A 553-bp *inv* DNA fragment (−328 to + 225) encompassing the OmpR and RovA binding sites was used. Lane 1—*inv* promoter fragment incubated without proteins. DNA-protein complexes were separated by electrophoresis in 6% native polyacrylamide gels and silver stained. The arrowhead indicates the band excised for MS/MS analysis.

**Figure 10:**
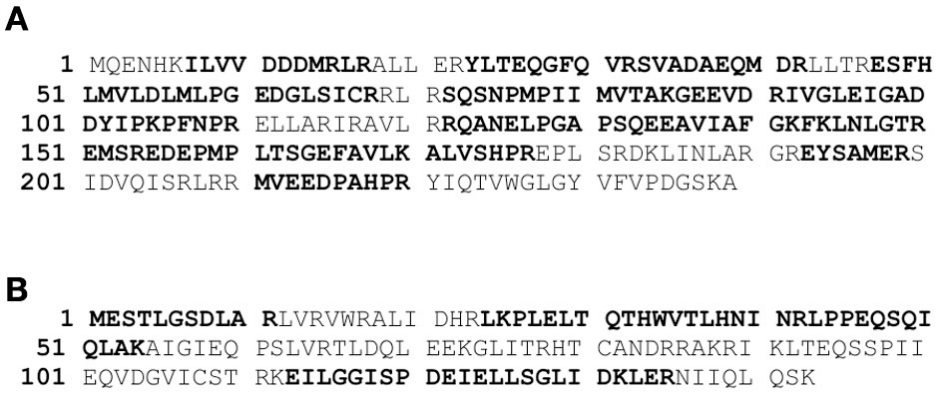
**Amino acid sequencing and bioinformatic analysis of proteins identified by the LC-MS/MS.** The DNA-protein complexes (indicated by the arrowhead at Figure 9) were subject to the MS/MS analysis. MS/MS data were used to search protein database. **(A)** The amino acid sequences of OmpR *Y. enterocolitica* strain Ye9 (GI 28912448) and RovA *Y. enterocolitica* 8081(GI 123442405) derived from the NCBI database **(B)**. Peptides detected by MS/MS analysis are indicated in bold. Sixty-nine percentage of OmpR and forty-five percentage of RovA protein sequence are covered by matching peptides.

EMSAs to examine competition between RovA and OmpR for binding to the *inv* regulatory region tested the effect of the order of binding, i.e., RovA added to the DNA before OmpR and *vice versa* (Figure 9). Initially, the 553-bp *inv* promoter fragment was first incubated with OmpR and then increasing amounts of RovA were added to the binding reaction (Figure 9, lanes 2–5). The OmpR protein, once bound to DNA, was not modified or displaced by RovA, since specific RovA-DNA complexes were not observed. In the inverse reactions, incubation of the *inv* promoter fragment first with RovA followed by the addition of increasing amounts of OmpR resulted in the disappearance of RovA-DNA complexes. In addition, a slower migrating band appeared at a lower concentration of OmpR (Figure 9, lane 7). To determine whether RovA is part of this slow mobility complex, the slice of gel containing the shifted band from EMSA, visualized by ethidium bromide staining, was subjected to LC-MS/MS analysis (Figure 10). Mass spectrometry identified two types of proteins: OmpR, classified according to the protein database of NCBI as the osmolarity response regulator of *Y. enterocolitica* and RovA, classified as the transcriptional regulator SlyA (RovA is a member of Mar/SlyA family). Thus, using this approach we were able to demonstrate the co-migration of OmpR and RovA to the same region of the gel during *in vitro* EMSA. However, these results do not necessarily imply simultaneous binding of these two proteins. The presented results are an initial attempt to gain some insight into the mechanism of RovA/OmpR interplay and additional experiments need to be performed to confirm this hypothesis.

## Discussion

In enteropathogenic *Yersinia*, signals from the environment seem to play a crucial role in the control of *inv* synthesis by engaging a number of activator and repressor proteins which together form a complex regulatory system (Ellison et al., 2004; Ellison and Miller, 2006b; Carlsson et al., 2007). Most investigations of *Y. enterocolitica inv* have been performed using high-pathogenicity *Y. enterocolitica* strain 8081 v of bioserotype 1B/O8. Recent intensive genetic and physiological studies have revealed significant differences in the pattern of *inv* synthesis between different serotypes and strains (high- and low-pathogenicity bioserotypes) in response to conditions including temperature and growth phase. While the expression of *inv* in *Y. enterocolitica* serotype O8 was high at moderate temperature, dramatically reduced at 37°C, and up-regulated at 37°C under low pH conditions, its expression in serotype O3 strains was found to be constitutive and significantly enhanced due to an IS insertion that provides specific activating elements (Uliczka et al., 2011). Analysis of the *inv* promoter activity using the *luxCDABE* reporter system revealed differences in *inv* expression between strains of serotypes O8 and O9 (Trček et al., 2010). However, neither specific regulatory factors nor the structure of the respective *inv* promoter regions seem to be responsible for the observed differences. Furthermore, in light of the data from these studies, it is possible that specific regulation of *inv* expression occurs at the single cell level.

Our previous study performed with *Y. enterocolitica* strain Ye9 (serotype O9) showed a significant decrease in *inv* expression at 37°C at neutral pH, similar to that seen in *Y. enterocolitica* serotype O8 (Brzostek et al., 2007). In the present study, low pH-dependent *inv* gene induction was demonstrated at both 25°C and 37°C in strain Ye9, which is different from the pattern of *inv* transcription observed previously in serotype O8 (Pepe et al., 1994) Therefore, genetic variation in separate bio-serotypes of *Y. enterocolitica* may lead to differences in *inv* expression.

The OmpR protein is a response regulator of the EnvZ/OmpR TCS that senses osmolarity and pH conditions (Aiba et al., 1989; Bang et al., 2000). In *Y. enterocolitica* Ye9, OmpR negatively affects *inv* transcription at moderate temperature and neutral pH (Brzostek et al., 2007). Studies on related EnvZ/OmpR signaling pathways in other enteric bacteria have shown that the modulation of gene expression is often mediated by OmpR regulatory proteins, which are themselves tightly regulated (Huang et al., 1992; Bang et al., 2000, 2002). Thus, besides the functional state of OmpR (i.e., the level of phosphorylation), changes in OmpR expression mediated by environmental signals could influence *inv* gene transcription. In the present study, the pattern of *ompR* transcription in *Y. enterocolitica* Ye9 exhibited marked differences in response to changes in temperature (reduced expression at 37°C vs. 25°C). In addition, a marked increase in *ompR* transcription was observed at pH 5.5 compared with pH 7.0, highlighting the acid-induced nature of *ompR* expression, as previously described in *Y. pestis* and *Salmonella enterica* (Hu et al., 2009a; Gao et al., 2011). These changes in expression, demonstrated at the mRNA level, were also observed at the protein level when cytoplasmic OmpR was evaluated by Western blotting. It is intriguing that *inv* transcription increases under conditions where the highest levels of *ompR* induction occur (at pH 5.5). Thus, we cannot rule out the possibility that under these environmental conditions, OmpR could positively modulate the expression of *inv* through interactions with other putative *inv* regulators implicated in pH-dependent regulation. This phenomenon is currently being investigated in greater detail.

To learn more about the involvement of OmpR in the *inv* regulatory circuit of *Y. enterocolitica* strain Ye9—in which RovA should play a major role—we evaluated the level of *inv* transcription in *ompR*, *rovA*, and *ompR rovA* mutant backgrounds. As anticipated, in the presence of RovA, the lack of OmpR led to an increase in *inv* transcription. However, in the *rovA* mutant background, this effect was no longer visible. These data showed that the inhibitory effect of OmpR on *inv* expression can be observed when RovA is present/active in *Y. enterocolitica* cells and suggest that, RovA does not act as a derepressor of OmpR *inv* inhibition. However, this scenario is complicated by the fact that in the absence of RovA, another regulatory protein, namely H-NS, probably operates as the main repressor of *inv* expression and overcomes the repressive activities of OmpR under the tested growth conditions. H-NS has previously been shown to contribute to the inhibition of *inv* expression, either alone (*Y. pseudotuberculosis*) or together with YmoA (*Y. enterocolitica*) (Heroven et al., 2004; Ellison and Miller, 2006b). Thus, our results suggest that OmpR might influence *inv* expression by inhibiting RovA-dependent *inv* activation. It has previously been shown that RovA, besides its anti-H-NS repressor activity, might directly stimulate *inv* transcription. This type of RovA activity, leading to increased *inv* transcription, has been observed in *Y. pseudotuberculosis* (Tran et al., 2005). The lack of success in obtaining a *Yersinia hns* mutant led to the construction of a heterologous system in *E. coli*, which has been used to study the regulatory role of the H-NS protein (Heroven et al., 2004; Ellison and Miller, 2006b). Using this approach, we recently demonstrated a marked increase in *inv* expression in *E. coli* strains carrying a mutation in either the *hns* or *ompR* genes. Moreover, our studies using an *E. coli hns* strain indicated that the RovA protein of *Y. enterocolitica* O9 may act as an activator of *inv* expression, while OmpR seems to repress the RovA-dependent activation of the *inv* gene (Raczkowska et al., 2011a).

To study the nature of the interactions of RovA and OmpR with the *inv* promoter region in *Y. enterocolitica* O9 and to characterize the interplay between these regulatory proteins, we performed DNA mobility shift assays. When used separately in EMSAs, OmpR and RovA could bind specifically to an *inv* DNA fragment comprising the putative OmpR and RovA binding sites. These assays suggested that OmpR binds to a unique site within the *inv* promoter, confirming our previous findings and the results of *in silico* analysis (Brzostek et al., 2007). In contrast, the binding properties of RovA raised the possibility of the presence of more than one RovA binding site within the *inv* promoter. Two potential RovA binding sites (low and high affinity) were previously identified in the *inv* promoter of *Y. enterocolitica* and *Y. pseudotuberculosis*, and it has been suggested that optimal expression of *inv* depends on the interaction of RovA molecules with these sequences (Nagel et al., 2001; Heroven et al., 2004; Ellison and Miller, 2006b). In competitive DNA mobility shift assays, the addition of increasing concentrations of RovA to a constant amount of OmpR already bound to the *inv* promoter fragment, did not lead to changes in the mobility of the nucleoprotein complexes. However, in the opposite scenario, the disappearance of RovA-DNA complexes was observed upon the addition of increasing amounts of OmpR and this was accompanied by the appearance of a major protein-DNA complex of reduced mobility. Mass spectrometry analysis revealed the presence of both the RovA and OmpR proteins in this slower migrating complex. This co-migration may indicate the simultaneous binding of these proteins at the *inv* promoter (the OmpR and RovA recognition sites in the *inv* promoter do not overlap). Simultaneous binding of both regulators might reflect a potential mechanism of RovA/OmpR interplay influencing *inv* expression. However, our data are preliminary in nature and confirmation of this hypothesis awaits more detailed experiments including supershift assays with specific OmpR and RovA antibodies and DNA footprinting with DNA fragments lacking the predicted binding sites. Such studies may shed light on the mechanism of RovA/OmpR interplay and the possibility that activated OmpR might bind to its specific binding site even in the presence of RovA protein, or that binding of OmpR may disturb RovA binding (at the low affinity site located near the putative OmpR binding site). The presence of two RovA binding sites within the *inv* promoter, of high and low affinity, may permit fine-tuning of *inv* expression by RovA, OmpR and other regulatory proteins that interact with the *inv* regulatory sequence.

In addition, our EMSA data indicated that phosphorylation of OmpR by acetyl-phosphate may result in its activation, leading to slight enhancement of its binding abilities.

OmpR phosphorylation, by acetyl-phosphate and other phospho-donors or related kinases, has previously been suggested for *E. coli*, (Forst et al., 1990; McCleary and Stock, 1994; Shin and Park, 1995; Matsubara and Mizuno, 1999) and for *Y. enterocolitica* (Raczkowska et al., 2011b).

As a final experiment to characterize the regulation of *inv* gene expression, a *rovA*::*lacZYA* chromosomal fusion was used to examine whether the OmpR regulator could influence *rovA* transcription. Our results indicated that OmpR has no effect on *rovA* transcription, which suggests that the influence of OmpR on *inv* expression does not occur through modulation of RovA levels. These data also showed significant differences in the activity of the *rovA* promoter in *Y. enterocolitica* Ye9 cells grown at different temperatures. Expression of RovA in response to conditions including temperature and growth phase is important for the environmentally-controlled expression of *inv* in enteropathogenic *Yersinia* (Nagel et al., 2001; Heroven et al., 2004; Ellison and Miller, 2006b). We found lower levels of *rovA* expression at 37°C compared with 25°C. However, our data showed only a 2-fold decrease in the activity of the *rovA* promoter in *Y. enterocolitica* Ye9 cells grown at 37°C compared to 25°C, which contrasts with similar data for *Y. enterocolitica* O8 and *Y. pseudotuberculosis*, showing that the levels of *rovA* transcription were reduced by 4-fold at the higher temperature (Nagel et al., 2001; Lawrenz and Miller, 2007). Thus, the precise mechanism of thermoregulation of *inv* governed by RovA protein might be different in high- and low-pathogenicity bioserotypes of *Y. enterocolitica*. The regulation of *rovA* in *Y. pseudotuberculosis* is mediated by the H-NS and RovA proteins, and probably follows the mechanism proposed for the *inv* gene. The available data suggest that, similarly to the regulation of *inv* gene expression, the relative levels of RovA and H-NS could be responsible for controlling *rovA* expression. However, the regulation of *rovA* in *Y. enterocolitica* may be less straightforward, with the latest findings indicating the possible indirect involvement of RovA in regulating *rovA* expression (Lawrenz and Miller, 2007). In addition, a third protein named RovM, a LysR-type regulator, has been shown to negatively modulate *rovA* expression in both enteropathogenic species (Heroven and Dersch, 2006). Furthermore, the Crs system has been found to affect expression of the *rovA* gene by regulating RovM synthesis (Heroven et al., 2008). In addition to H-NS, RovA and RovM, a fourth regulator of *rovA*, named LeuO, has also been identified. This LysR-like regulator appears to positively affect the expression of *rovA* in *Y. enterocolitica* (Lawrenz and Miller, 2007). Recently, RovA was identified as a putative protein thermometer. Thermal shifts from 26°C to 37°C probably lead to reversible conformational changes in RovA, which reduce its DNA-binding functions and render it more susceptible to proteolysis (Herbst et al., 2009).

In summary, our results indicate that OmpR in *Y. enterocolitica* serotype O9 directly influences *inv* expression via binding to the *inv* promoter, but not through modulation of *rovA* expression. In addition, phosphorylation of OmpR by acetyl-P appears to stimulate its binding ability. However, the mechanism by which phosphorylated OmpR represses the expression of *inv* remains unknown. Our findings raise the possibility that OmpR-P binding to the *inv* promoter could influence RovA interaction with two binding sites of different affinities located in this region.

### Conflict of interest statement

The authors declare that the research was conducted in the absence of any commercial or financial relationships that could be construed as a potential conflict of interest.
